# Supplementary figures and images for: Analysing microbiome intervention design studies: Comparison of alternative multivariate statistical methods
Source: PLoS One. 2021 Nov 18;16(11):e0259973. doi: 10.1371/journal.pone.0259973 (PMC8601541; doi:10.1371/journal.pone.0259973)

(A) Moen data set

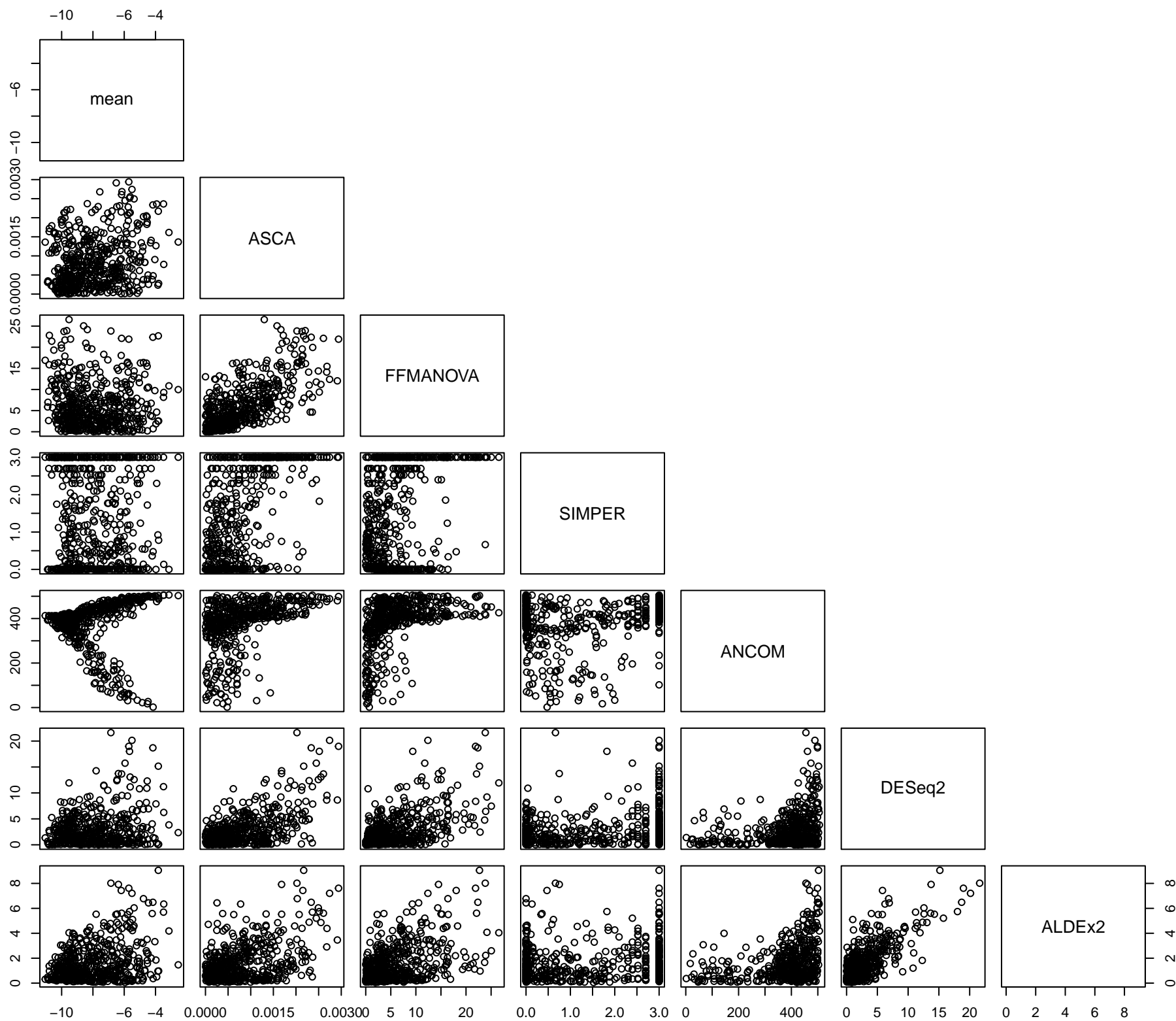

(B) Lai data set

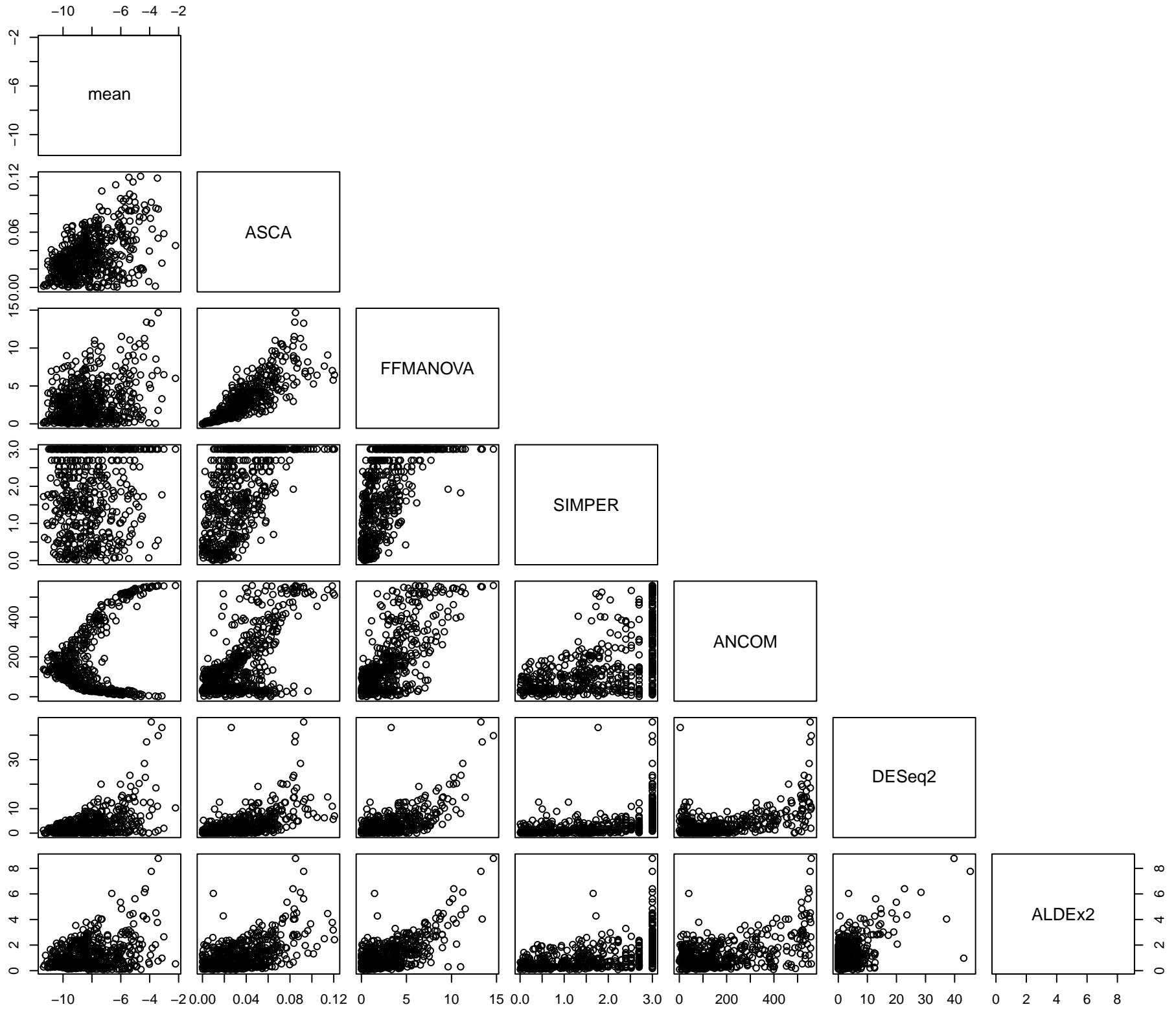

(C) Le Sciellour data set

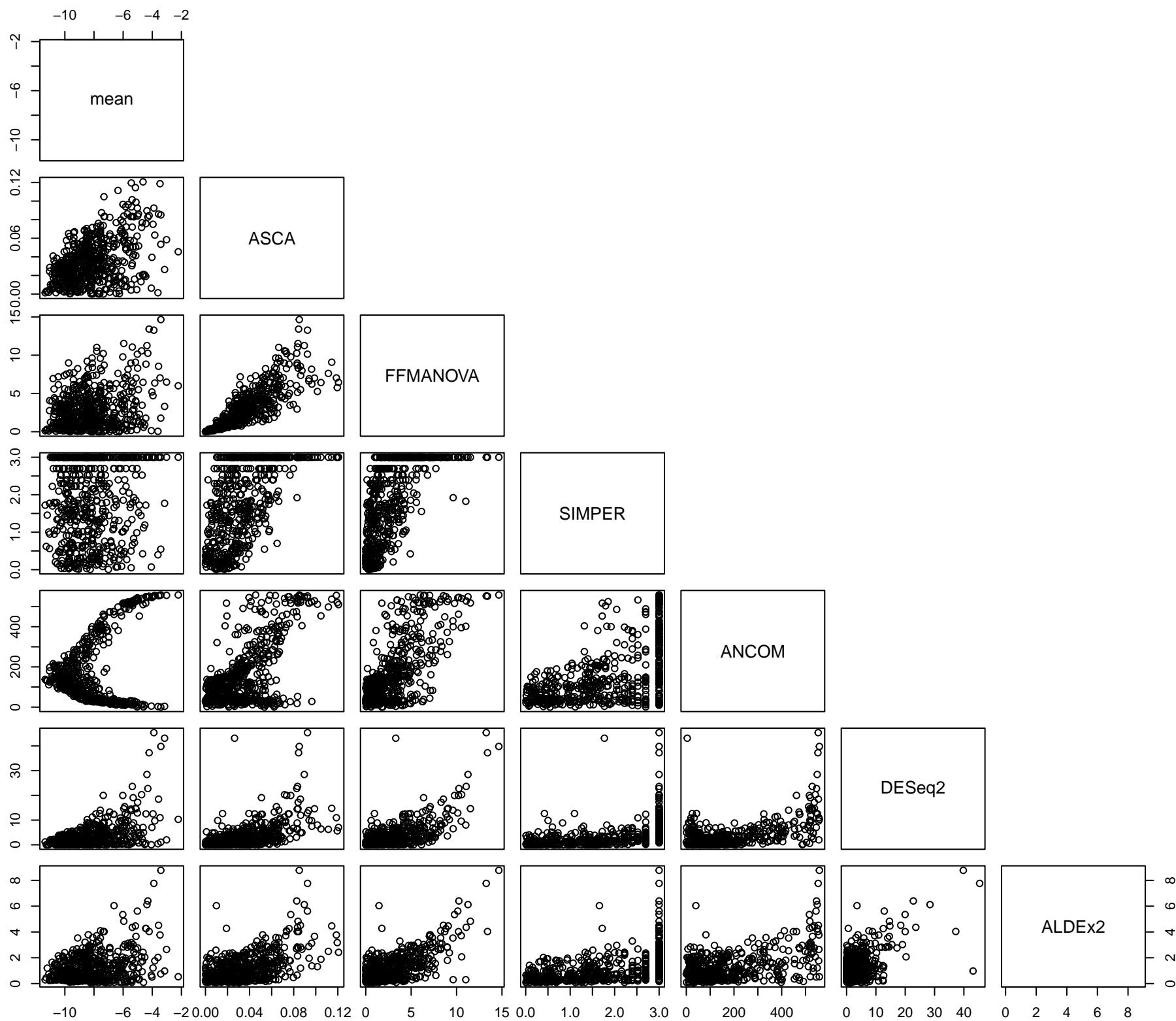

(D) Wang data set

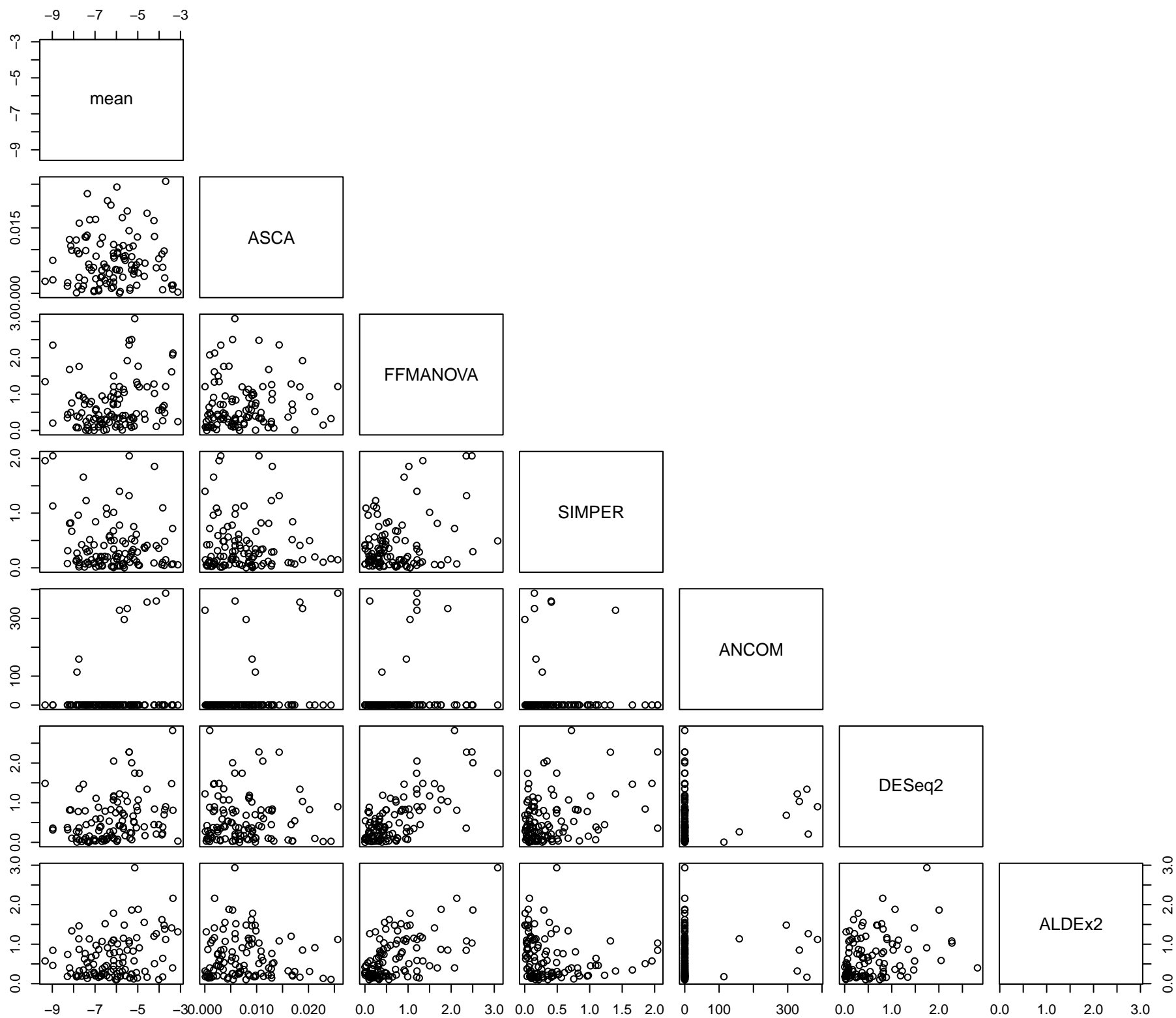

(E) Birkeland data set

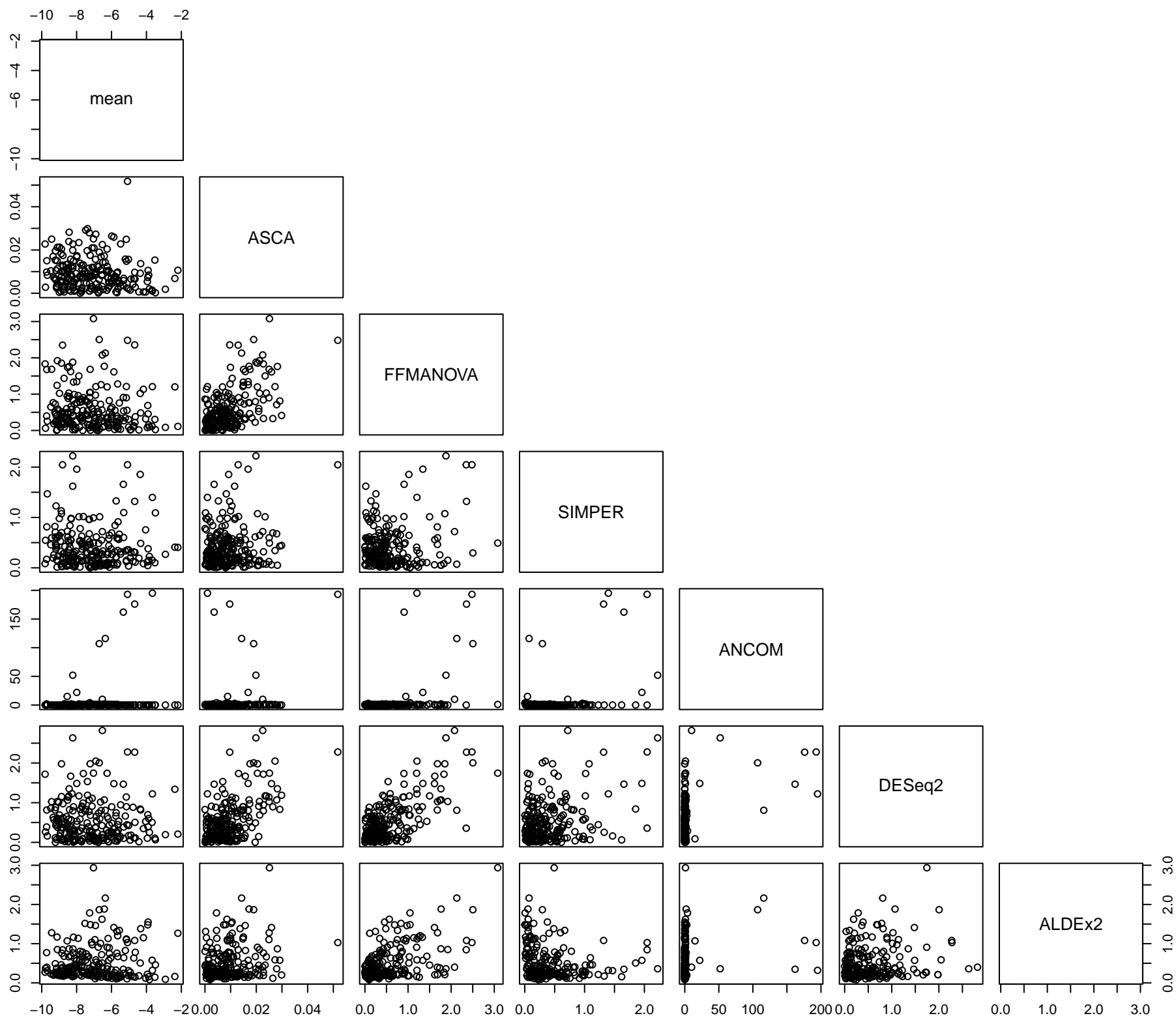

Supplement: S1 Fig — The (A) Moen, (B) Lai, (C) Le Sciellour, (D) Wang and (E) Birkeland data sets. (PDF) [file pone.0259973.s001.pdf]

# Few\_Low

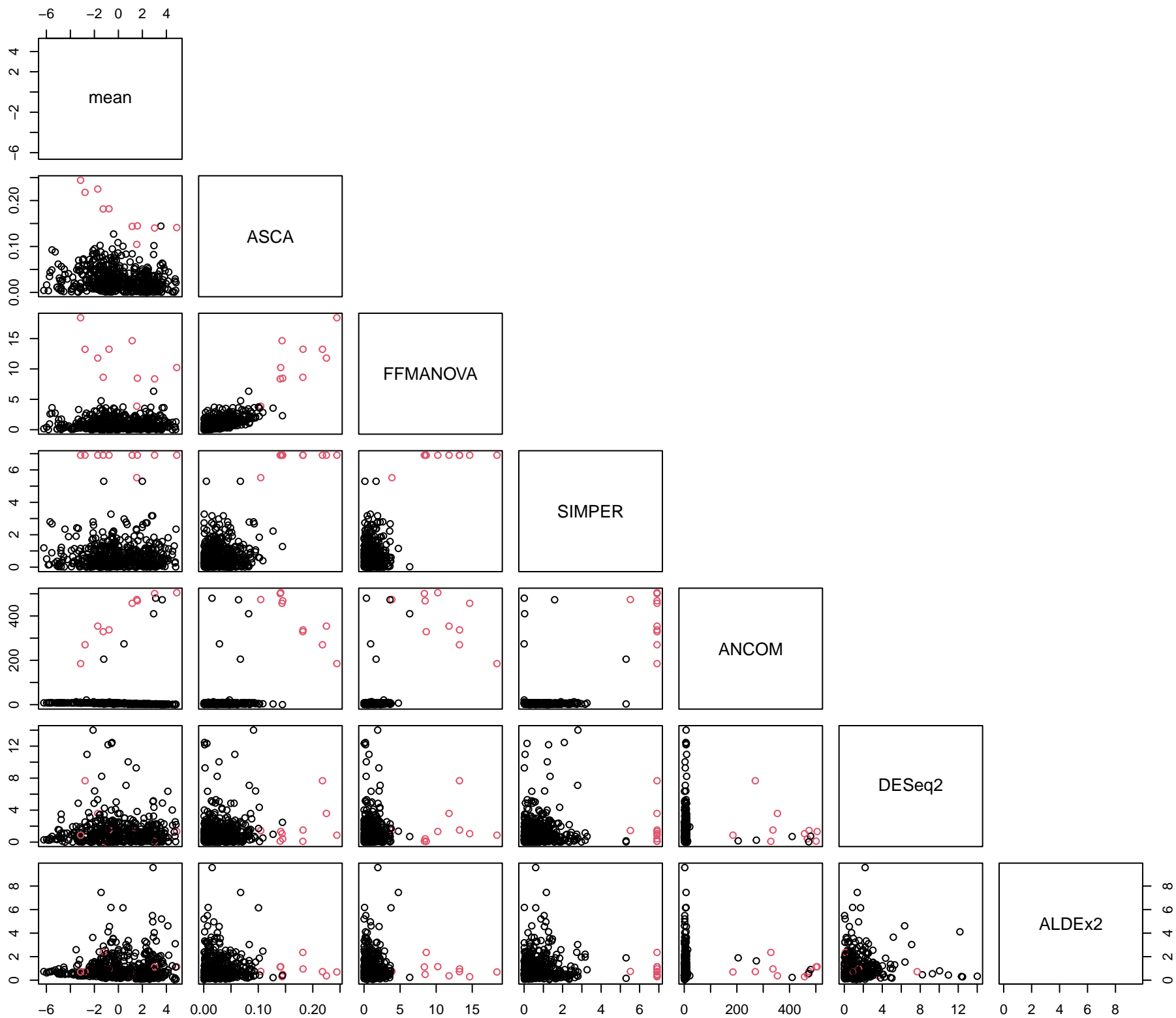

# Few\_High

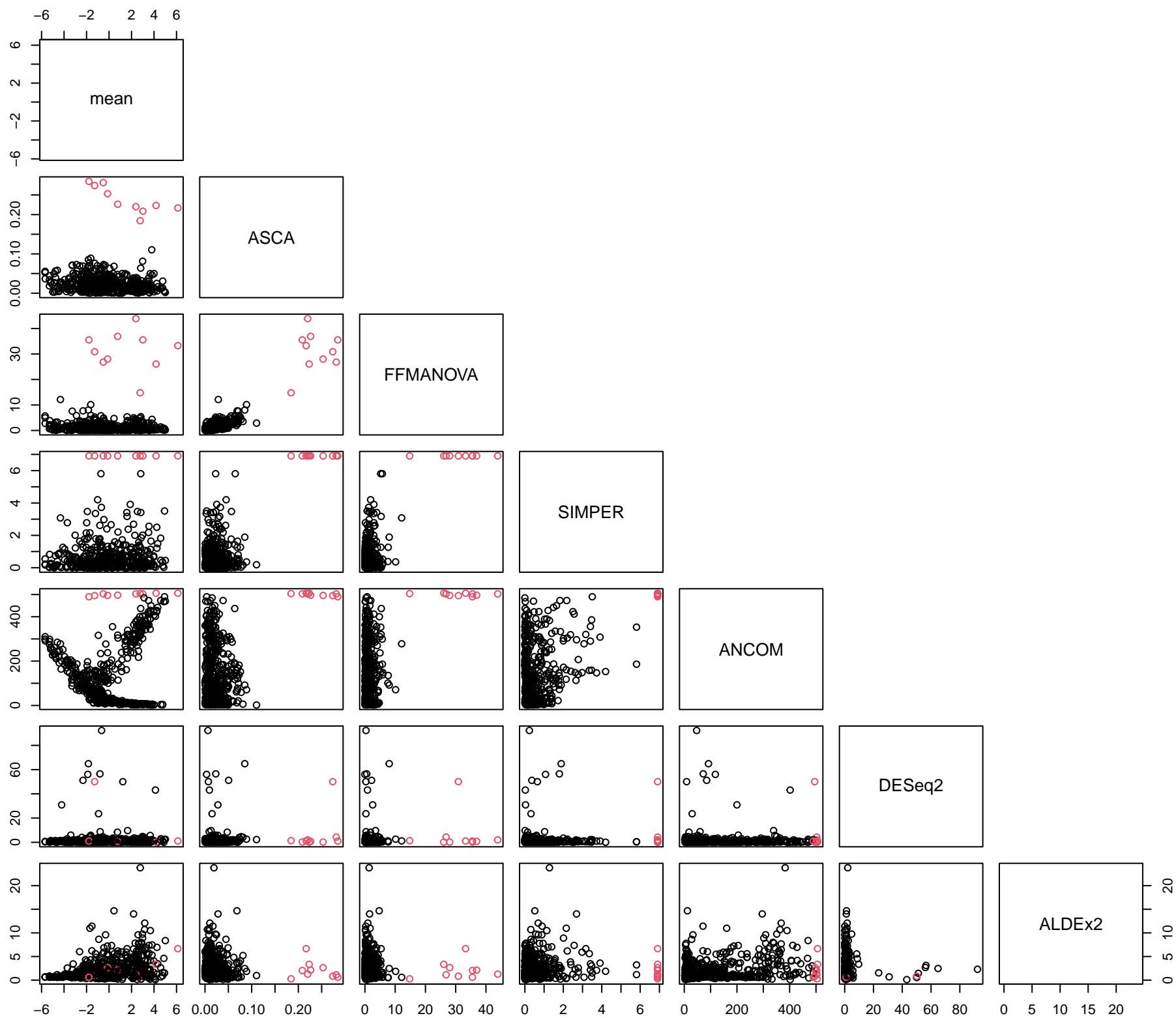

# Many\_Low

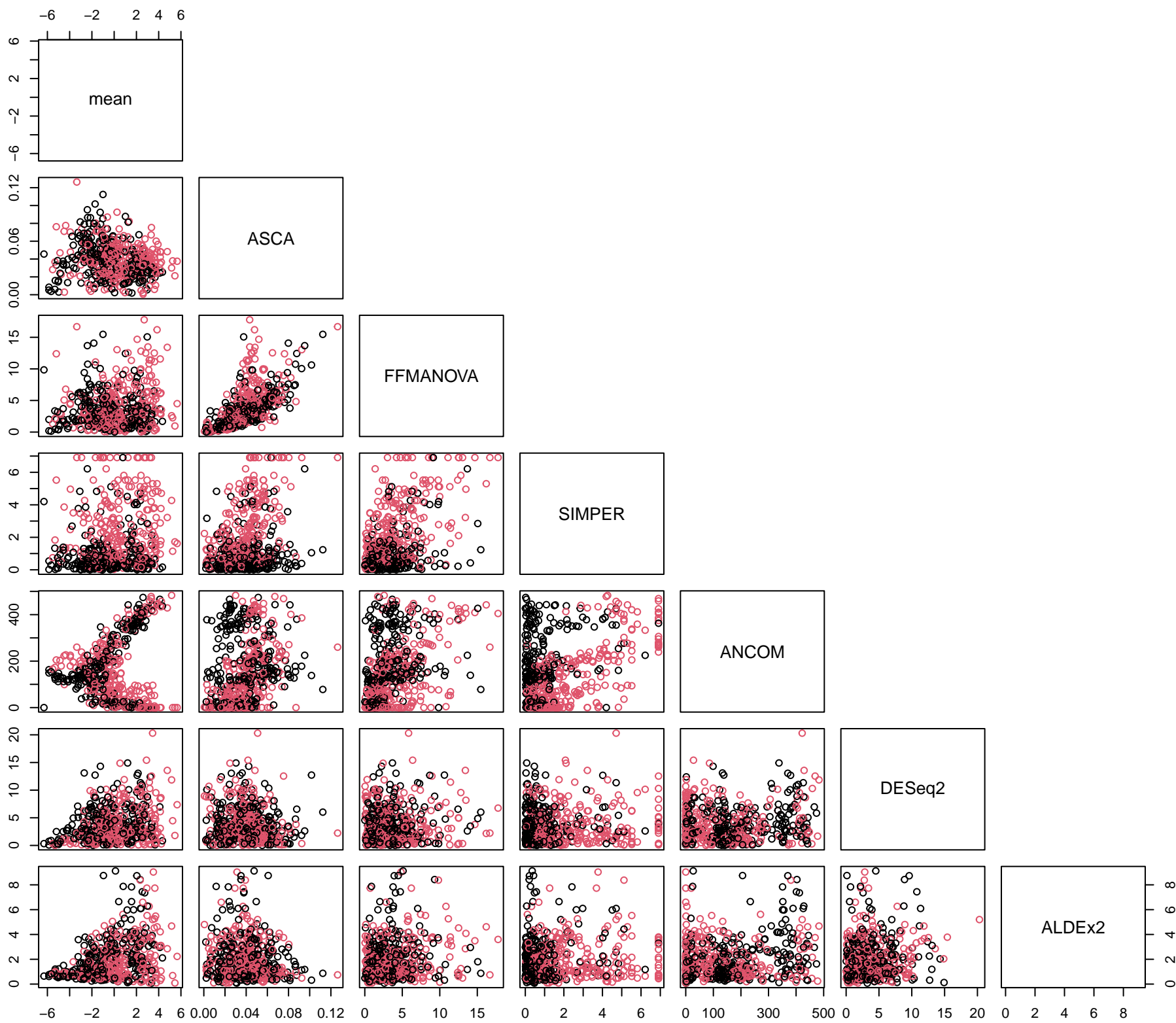

# Many\_High

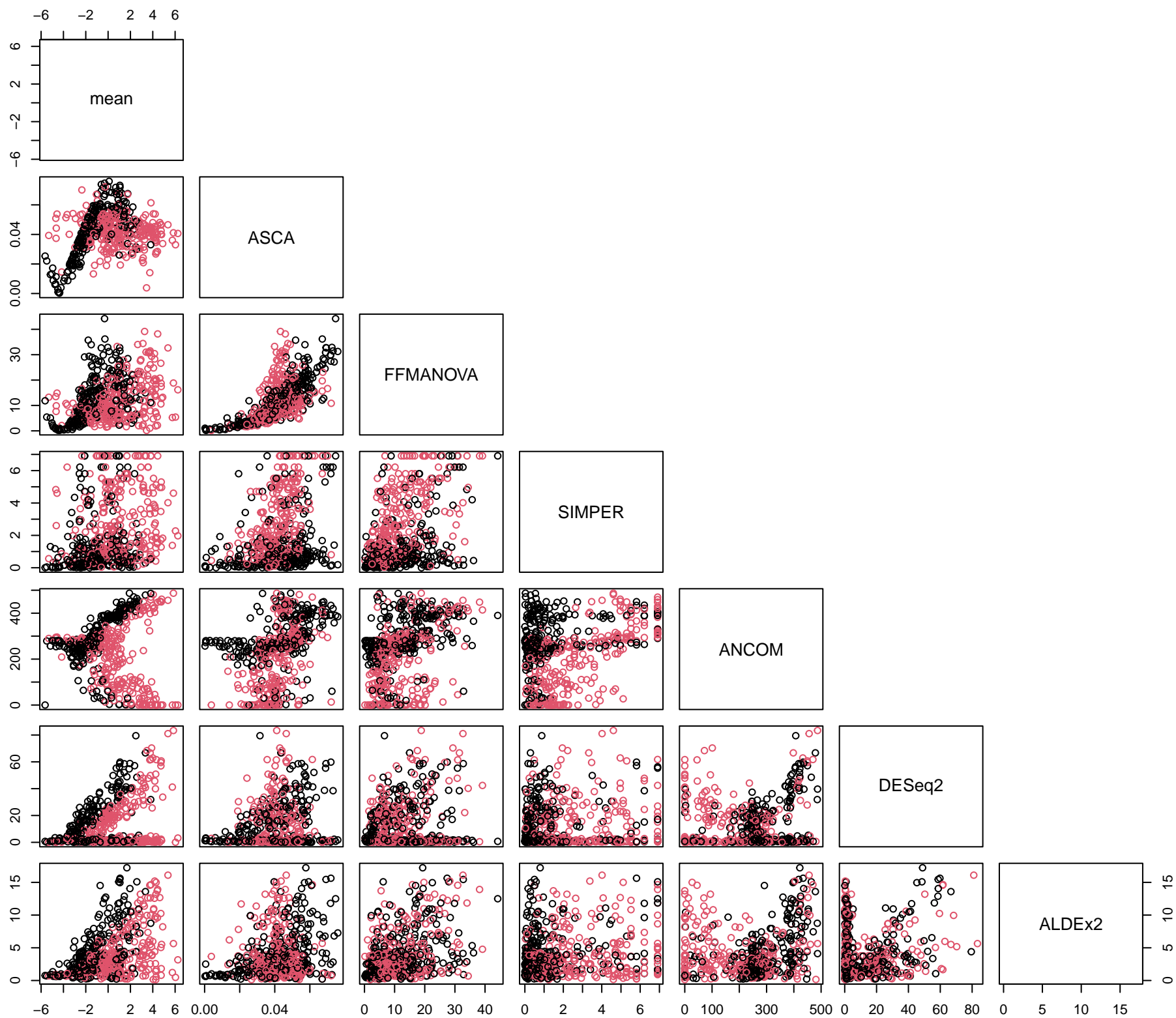

Supplement: S2 Fig — Differentially abundant OTUs are shown in red and non-differentially abundant OTUs in black. (PDF) [file pone.0259973.s002.pdf]
