# Supplementary material for: Analysing microbiome intervention design studies: Comparison of alternative multivariate statistical methods
Source: PLoS One. 2021 Nov 18;16(11):e0259973. doi: 10.1371/journal.pone.0259973 (PMC8601541; doi:10.1371/journal.pone.0259973)
Supplement: Attachment — Submitted filename: Comments.pdf [file pone.0259973.s012.pdf]

Referee Report on "Analysing microbiome intervention design studies: Comparison of alternative multivariate statistical methods"

by Maryia Khomich, Ingrid Mage, Ingunn Berget and Ida Rud

In this article, the authors conducted a detailed comparison between different statistical methods for microbiome data analysis. The comparison is based on some dietary intervention studies. I enjoy reading this paper and think such a comparison is needed. But there is still a large room to improve in numerical comparison and writing.

Major Points

- Several methods suggesting the same conclusion doesn't imply that they are reliable. All methods may be wrong in the same way! It might be better to benchmark the analysis with some special experiment design or simulation study on real data.
- In the OTU level comparison, several other popular differential abundance tests should be also included into the comparison. For example, DESeq2[4], MetagenomeSeq [7], ANCOM-BC[3], DR [5].
- In distance based method, the choice of distance is very important. It would be great to compare different choices of distance.
- In abundance-based methods, phylogenetic tree information can also be incorporated [6, 9]. They should be cited.
- In most of abundance based method, the zeros are usually replaced by some small positive constant. This is a very important problem in microbiome data analysis. Currently, the authors only consider one imputation method. It would be better to include a separate experiment to compare different ways to handle zeros. Some studies [2] suggests that handling zeros can affect the analysis a lot. Some recent statistical methods [1, 8] are designed for zero problem should be at least cited.

References

[1] B. Brill, A. Amir, and R. Heller. Testing for differential abundance in compositional counts data, with application to microbiome studies. *arXiv preprint arXiv:1904.08937*, 2019.

[2] P. I. Costea, G. Zeller, S. Sunagawa, and P. Bork. A fair comparison. *Nature methods*, 11(4):359–359, 2014.

[3] H. Lin and S. D. Peddada. Analysis of compositions of microbiomes with bias correction. *Nature communications*, 11(1):1–11, 2020.

[4] M. I. Love, W. Huber, and S. Anders. Moderated estimation of fold change and dispersion for rna-seq data with deseq2. *Genome biology*, 15(12):1–21, 2014.

[5] J. T. Morton, C. Marotz, A. Washburne, J. Silverman, L. S. Zaramela, A. Edlund, K. Zengler, and R. Knight. Establishing microbial composition measurement standards with reference frames. *Nature communications*, 10(1):1–11, 2019.

[6] J. T. Morton, J. Sanders, R. A. Quinn, D. McDonald, A. Gonzalez, Y. Vázquez-Baeza, J. A. Navas-Molina, S. J. Song, J. L. Metcalf, E. R. Hyde, et al. Balance trees reveal microbial niche differentiation. *MSystems*, 2(1), 2017.

[7] J. N. Paulson, O. C. Stine, H. C. Bravo, and M. Pop. Differential abundance analysis for microbial marker-gene surveys. *Nature methods*, 10(12):1200–1202, 2013.

[8] S. Wang. Robust differential abundance test in compositional data. *arXiv preprint arXiv:2101.08765*, 2021.

[9] S. Wang, T. T. Cai, and H. Li. Hypothesis testing for phylogenetic composition: a minimum-cost flow perspective. *Biometrika*, 2020.
